# How to Improve Sensitivity of Sandwich Lateral Flow Immunoassay for Corpuscular Antigens on the Example of Potato Virus Y?

**DOI:** 10.3390/s18113975

**Published:** 2018-11-15

**Authors:** Shyatesa C. Razo, Vasily G. Panferov, Irina V. Safenkova, Yuri A. Varitsev, Anatoly V. Zherdev, Elena N. Pakina, Boris B. Dzantiev

**Affiliations:** 1 A.N. Bach Institute of Biochemistry, Research Centre of Biotechnology of the Russian Academy of Sciences, Leninsky Prospect 33, 119071 Moscow, Russia; kish218@gmail.com (S.C.R.); panferov-vg@mail.ru (V.G.P.); saf-iri@yandex.ru (I.V.S.); zherdev@inbi.ras.ru (A.V.Z.); 2 Agricultural-Technological Institute, RUDN University, Miklukho-Maklaya Street 8/2, 117198 Moscow, Russia; e-pakina@yandex.ru; 3 A.G. Lorch All-Russian Potato Research Institute, Kraskovo, Lorch Street 23, 140051 Moscow, Russia; varyuriy@yandex.ru

**Keywords:** gold nanoparticles, increase of sensitivity, lateral flow test strips, potato virus Y, pre-incubation, sandwich immunoassay

## Abstract

A simple approach was proposed to decrease the detection limit of sandwich lateral flow immunoassay (LFIA) by changing the conditions for binding between a polyvalent antigen and a conjugate of gold nanoparticles (GNPs) with antibodies. In this study, the potato virus Y (PVY) was used as the polyvalent antigen, which affects economically important plants in the *Solanaceae* family. The obtained polyclonal antibodies that are specific to PVY were characterized using a sandwich enzyme-linked immunosorbent assay (ELISA) and surface plasmon resonance (SPR). For LFIA, the antibodies were conjugated with GNPs with a diameter of 17.4 ± 1.0 nm. We conducted LFIAs using GNP conjugates in a dried state on the test strip and after pre-incubation with a sample. Pre-incubating the GNP conjugates and sample for 30 s was found to decrease the detection limit by 60-fold from 330 ng∙mL^−1^ to 5.4 ng∙mL^−1^ in comparison with conventional LFIA. The developed method was successfully tested for its ability to detect PVY in infected and uninfected potato leaves. The quantitative results of the proposed LFIA with pre-incubation were confirmed by ELISA, and resulted in a correlation coefficient of 0.891. The proposed approach is rapid, simple, and preserves the main advantages of LFIA as a non-laboratory diagnostic method.

## 1. Introduction

Lateral flow immunoassay (LFIA) is the main tool used for non-laboratory diagnostics in many fields, including medicine, agriculture, environmental monitoring, and food quality control [1,2,3,4]. LFIA is based on the use of a composite of several membranes fixed on a support (i.e., the test strip). In general, immunoreagents (i.e., the antibodies in the test and control zones and their labeled conjugate) are provided in dry form. The immersion of one end of the test strip in the analyzed sample initiates its migration along the strip as well as the formation of a colored complex of immunoreactant and label in a certain zone of the strip [5,6]. The sequence and efficiency of the binding of the antigen with antibodies and conjugates depend on the kinetic conditions of the LFIA. The main advantage of LFIA is that it provides a rapid analysis in the absence of equipment and specially trained personnel. However, the main limitation of most LFIAs is associated with their high limit of detection (LOD) [7,8,9].

A high detection limit is caused by two main factors. First, a high detection limit is related to the limitations of label detection, such as gold nanoparticles. In this case, the task can be solved using various amplification tools, such as a pre-concentration of the sample, the aggregation or catalytic increase of the label size on the test strip, or the use of new types of labels [10,11,12,13]. Second, the incomplete binding of antibodies with the antigen under the kinetic conditions of the LFIA causes a high detection limit. Improvement is possible by isolating one of the interactions (the most common is the interaction of an antigen and an antibody: a label conjugate) at a particular homogeneous stage when the two interacting reagents are well mixed before moving through the membrane. In such homogeneous stages, the frequency of effective collisions increases, and chemical equilibrium is quickly reached. An example of this approach is the pre-incubation of a gold nanoparticle (GNP) conjugate with a sample.

Pre-incubation was successfully used in a competitive format of LFIA for the detection of veterinary drug residues (clenbuterol, sulfadiazine, and tetracycline) [14], the aptamer-based detection of β-conglutin [15] and aflatoxin B1 [16], and the simultaneous detection of five chemicals in drinking water [17]. Dentg et al. showed that the LOD decreased by 3.5 times in clenbuterol detection (i.e., using five minutes pre-incubation) in comparison with the conventional LFIA [18]. Zvereva et al. presented a LOD that decreased by 14 times in ractopamine detection [19], which confirmed the pre-incubating approach. There are some commercial lateral flow tests using pre-incubation, such as rapid tests for the detection of mycotoxins and antibiotics of Unisensor (Belgium, www.unisensor.be), AuroFlow strip tests for the detection of antibiotics in milk (Bioo Scientific Corporation, Austin, TX, USA, www.biooscientific.com), and cryptococcal antigen lateral flow assay of Hain Lifescience, SA (South Africa, www.hain-lifescience.co.za). However, attempts to apply a similar pre-incubating approach in sandwich assay were very few and lacked comparison with conventional sandwich LFIA. Moreover, only prolonged incubations were used in these works, and the total duration of the assays increased significantly. Previous studies conducted an aptamer-based LFIA for adenosine triphosphate detection with a 10-min pre-incubation time [20]; an LFIA for the detection of autoantibodies to tyrosine phosphatase-like protein IA-2 with a 30-min pre-incubation time [21]; an LFIA with a 10-min pre-incubation of antibody—magnetic nanoparticle conjugate—and a sample for the detection of potato virus X [22]. When large polyvalent antigens, such as viruses and bacteria, are used, the interaction with GNP–antibody conjugates in the stream through the membrane is complicated by a low diffusion coefficient. However, no previous study has focused on the improvement of LFIA by the preliminary mixing of reagents in sandwich LFIAs, which are typical for polyvalent antigens.

This study is the first to propose the use of a short pre-incubating approach with the sandwich LFIA for detection of a polyvalent viral antigen. Compared with the competitive format, the sandwich format has more complex kinetic processes, because a ternary complex is formed in the test zone, including an antibody immobilized on the membrane, an antigen, and an antibody conjugate with a detectable label [5,23,24]. The proposed approach is based on the pre-mixing of the GNP–antibody conjugate with the sample.

Potato virus Y (PVY) (family *Potyviridae*) was used as a target polyvalent analyte. PVY was selected as the viral antigen, because it is one of the important and dangerous plant viruses in the family *Solanaceae* [25,26]. Virions with a mass of more than 60 × 10^3^ kDa have filament-like structures, the length is 680–900 nm, and the diameter is 11–20 nm [27,28]. The virion consists of approximately 2000 copies of 33 kDa coat proteins (CP) and RNA enclosed inside a capsid formed by CP [28]. In potato, PVY can induce a range of different symptoms, the most common being a leaf mosaic. However, the virus can also cause more severe effects, and is the causal agent of potato tuber necrotic ringspot disease [25,26]. An infected plant with mild symptoms of infection usually contains PVY RNA copies in the range from 2.4 × 10^9^ per 1 mg of freeze-dried potato leaves [29], which is approximately 100 ng of PVY. This amount of PVY should be considered in developing test systems. Thus, the aim of the present study is to develop an LFIA for PVY detection based on the pre-mixing of the GNP–antibody conjugate and the sample, as well as compare the results with the conventional LFIA using dried conjugates implemented on some of the same reagents.

## 2. Materials and Methods

### 2.1. Reagents

Ordinary and necrotic types of potato virus Y (PVY^N^, PVY^O^), potato viruses X, M, S, A, and potato leafroll virus were obtained from the A.G. Lorch All-Russian Potato Research Institute (Moscow, Russia). Tris(hydroxymethyl)aminomethane (Tris), Triton X-100, 3,3′,5,5′-tetramethylbenzidine dihydrochloride (TMB), sodium azide, Tween-20, bovine serum albumin (BSA), chloroauric acid, N-hydroxysuccinimide (NHS), 1-ethyl-3(3-dimethylaminopropyl) carbodiimide hydrochloride (EDC), biotinamidohexanoyl-6-aminohexanoic acid N-hydroxysuccinimide ester, ethanolamine, and a conjugate of streptavidin-polyperoxidase were obtained from Sigma-Aldrich (St. Louis, MO, USA). Protein A derived from *Staphylococcus aureus* was purchased from Imtek (Moscow, Russia). All of the salts, acids, and solvents were of analytical reagent or chemical reagent grade. All of the solutions that were used to obtain the GNPs and their conjugates were prepared using Milli-Q water (Millipore, MA, USA).

The nitrocellulose membranes (CNPC-12μ) adhering to the surface of a laminated card, conjugate release matrix (PT-R5), sample pads (GFB-R4, 0.35), and absorbent pads (AP045) were obtained from Advanced Microdevices (Ambala Cantt, Haryana, India).

### 2.2. Plant Extract Preparation

We used both healthy and infected potato plants (*in vitro* culture) to prepare the leaf extracts. The leaf extracts were obtained using a mortar and pestle. The leaves were thoroughly homogenized in 50 mM of potassium phosphate buffer (pH 7.4) with 0.05 M of NaCl (PBS) and 0.05% Triton X-100 (PBST) (1 g potato leaves:10 mL PBST). For the calibration curves, PVYs (both PVY^N^, PVY^O^) were added to the healthy extract in different concentrations.

### 2.3. Production of Polyclonal Antibodies

PVYN was used for immunization. Chinchilla rabbits (females, four to five months old) were immunized via a subcutaneous injection with complete Freund’s adjuvant, and then two sequential subcutaneous injections with incomplete Freund’s adjuvant were carried out at two weekly intervals. The virus dose for any injections was 50 µg per animal. Blood was collected on days 7–12 after the last injection. Immunoglobulin G (IgG) was isolated from the antiserum by affinity chromatography on protein A-Sepharose CL-4B (Sigma-Aldrich, USA) according to the manufacturer’s instructions.

### 2.4. Biotinylation of Antibodies

Antibodies were biotinylated as described by Hermanson [30]. Biotinamidohexanoyl-6-aminohexanoic acid N-hydroxysuccinimide ester was used to attach the amine groups of the antibodies. The mixture of biotin reagents and antibodies at a molar ratio of 15:1 was incubated for 1 h at room temperature with continuous mixing. Dialysis was then performed in 1000-fold PBS for 4 h at room temperature to remove the unreacted biotin reagent.

### 2.5. Synthesis of Gold Nanoparticles

GNPs were synthesized using the Frens method [31] modified by Safenkova et al. [32]. Briefly, 1 mL of 1% HAuCl_4_ was added to 95 mL of deionized water and heated to the boiling point; then, 4 mL of 1% sodium citrate was added. The solution was boiled for 30 min, cooled, and stored at 4 °C.

### 2.6. Synthesis of GNP–Antibody Conjugates

The GNP–antibody conjugates were synthesized according to Safenkova et al. [32] with the following features: GNPs (optical density at 520 nm (OD_520_) = 1.0) adjusted to 9.5 and antibody specific to PVY^N^ in 10 mM Tris-HCl, pH 9.5. The final concentration of the antibody in conjugate solution was determined as 12 μg∙mL^−1^ by flocculation curves. The synthesized GNP–antibody conjugate was transferred to a 50-mM potassium phosphate buffer (pH 7.4) with 0.1 M of NaCl containing 0.25% BSA, 0.25% Tween 20, 1% sucrose, and 0.02% NaN_3_. The spectra of the GNPs and their conjugates were recorded by Biochrom Libra S60 Double Beam Spectrophotometer (Biochrom, Cambridge, UK).

### 2.7. Transmission Electron Microscopy (TEM)

The GNPs were adsorbed on a copper grid coated with a poly (vinyl formal) for 10 min. Images were obtained using a JEM CX-100 transmission electron microscope (Jeol, Tokyo, Japan) and analyzed by Image-Tool (UTHSCSA, San Antonio, TX, USA).

### 2.8. Dynamic Light Scattering (DLS)

The hydrodynamic size and zeta potential of the GNPs, GNP conjugates, PVY, and PVY–GNP conjugate immune complexes were measured using Zetasizer Nano (Malvern Panalytical, Malvern, UK). The temperature was stabilized at 25 °С, and the scattering angle was 173°. The statistical analysis was performed using the Malvern software ver. 7.11.

### 2.9. Constants Measurement of the Interactions between Antibodies and PVY

The surface plasmon resonance (SPR) method was used to determine the equilibrium and kinetic dissociation constants of the antibodies. The BIAcore X (GE Healthcare, Chicago, IL, USA) instrument was used as described by Safenkova et al. [32]. Immune complexes consisting of covalently immobilized capture antibody, PVY^N^, and the injected antibody were formed on the chip surface, as described below. The carboxyl groups on the surface of the CM5 chip were activated by 0.4 M of EDC and 0.1 M of NHS. The captured anti-PVY antibodies were covalently immobilized (50 μg∙mL^−1^, pH 4.5) on the chip for seven minutes (5 µL∙min^−1^). The remaining active groups were deactivated by ethanolamine (1 M, pH 8.8) for seven minutes. After that, PVY^N^ (10 µg∙mL^−1^) was injected at a rate of 10 µL∙min^−1^ for six min. Then, anti-PVY antibodies in concentrations from 50 nM to 330 nM were injected into a 10-mM HEPES buffer (pH 7.4) that contained 150 mM of NaCl and 0.005% Tween-20. After each antibody injection, the chip was regenerated by glycine buffer (10 mM, pH 2.0) for one minute to remove PVY^N^. The constants were determined at a stage of the interaction between the PVY^N^ and the injected antibody.

The data were processed using BIAevaluation ver. 4.1 (GE Healthcare, USA). For the fitting, we used the same ranges of association stage (from 10 s to 150 s) and range of dissociation stages (from 265 s to 340 s) at all of the concentration measurements. The closeness of fit is described by the statistical values of χ^2^. For all of the measurements, values of χ^2^ were below 10. Means and errors for each kinetic constant were provided from the analysis of experimental series with different concentrations of PVY^N^.

### 2.10. Sandwich ELISA

Sandwich ELISA was carried out according to Panferov et al. [33] with the following modifications: the captured antibodies were used at a concentration of 1 μg∙mL^−1^ in PBS; the viruses were at concentrations ranging from 0.45 ng∙mL^−1^ to 1000 ng∙mL^−1^; the biotinylated antibodies were at a concentration of 1 μg∙mL^−1^. After the completion of all of the stages, including the addition of streptavidin–polyperoxidase conjugate, TMB substrate, and stop solution (1 M H_2_SO_4_), the optical density at 450 nm (OD_450_) was measured.

The enzyme-linked immunosorbent assay (ELISA) was performed using a Thermo Electron WellWash 4 MK2 washer (Fisher Scientific, Hampton, NH, USA). The results were registered by a Zenyth 3100 microplate reader (Anthos Labtec Instruments, Salzburg, Austria). All the statistical processing and calculations were done using Origin Pro 9.0 (Origin Lab, Northampton, MA, USA). The LOD was determined using the three-sigma method. The cross-reactivity was calculated as the ratio between the midpoints (IC50) of the calibration curves of PVY^N^ and the cross-reactant (i.e., another virus) multiplied by 100%.

### 2.11. ELISA Test of Antibody and GNP–Antibody Conjugates

The ELISA test of the antibody and antibody–GNP conjugates was carried out according to Safenkova et al. [34] with the following modifications: PVY^N^ at a concentration of 0.5 μg∙mL^−1^ was immobilized in microplate wells at 37 °C for 2 h; after the washing steps with PBST, anti-PVY antibodies at concentrations ranging from 0.45 ng∙mL^−1^ to 1000 ng∙mL^−1^ (or serial dilutions of the GNP–antibody conjugates from 1:10 to 2:10^4^) were used. After the completion of all of the stages, including the addition of the anti-rabbit antibody–peroxidase conjugate, the TMB substrate, and the stop solution (1 M H_2_SO_4_), the optical density was measured at 450 nm (OD_450_).

### 2.12. ELISA of the Viruses in Potato Leaf Extracts

The potato leaf extracts were tested using ELISA kits for PVY^N^ (Test Potato, Kraskovo, Russia) according to the manufacturer’s protocols. To calculate the virus content, the linear range of the calibration curve was used.

### 2.13. Preparation of Lateral Flow Test Strips

**Figure 1:**
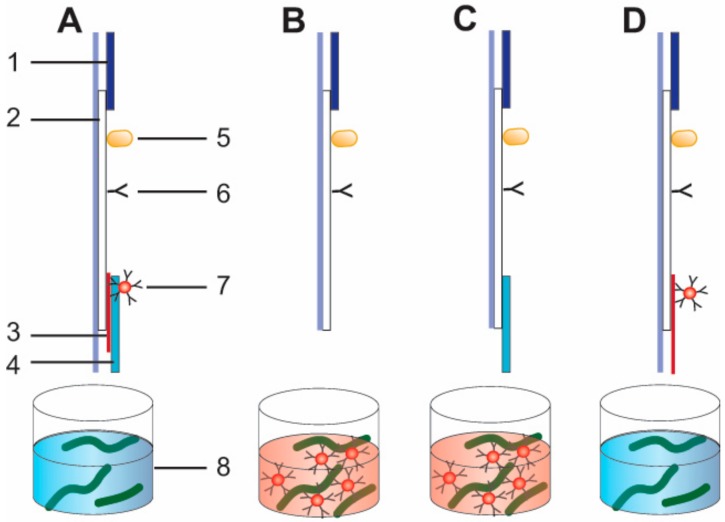
Schemes of lateral flow test strips. (**A**) Conventional lateral flow test strip with a conjugate pad. (**B**) Lateral flow test strip with pre-mixing and assembly without sample pad. (**C**) Lateral flow test strip with pre-mixing and assembly with sample pad. (**D**) Lateral flow test strip with a longer conjugate pad (10 mm). The numbers in the figure indicate (1) adsorbed pad, (2) plastic supports with the nitrocellulose membrane, (3) glass fiber membrane with conjugate, (4) sample pad, and (5) protein A immobilized on the control zone, (6) antibody immobilized on the test zone (7) gold nanoparticles (GNP)–antibody conjugate, and (8) sample with potato virus Y (PVY).

Test strips were prepared using plastic supports with the nitrocellulose membrane, glass fiber membrane with conjugate, adsorbed pad, and sample pad (Figure 1). Protein A at a concentration of 0.4 mg∙mL^−1^ in PBS with 5% glycerol was used for the control zone, and 1 mg∙mL^−1^ of antibodies specific to PVY were used for the test zone. Reagents were applied to the control and test zones of the nitrocellulose membrane by using an Isoflow Dispenser (Imagene Technology, Hanover, NH, USA). All of the reagents were dispensed at 0.15 mL per mm of membrane width. The nitrocellulose membranes were dried at 37 °C for 8 h. We used the four different test strip assemblies shown in Figure 1 to accomplish the tasks of the study.

For test strips with the conjugate pad (1) (see Figure 1A), the GNP–antibody conjugates were deposited onto glass fiber membranes from a solution of OD520 = 4; the conjugate load was 1.6 mL per mm of strip width. The glass fiber membranes were dried at room temperature for 8 h. Then, we attached glass fiber membranes, sample pads, and adsorbed pads to plastic supports with the nitrocellulose membrane.

For test strips with pre-mixing (2, 3), we attached only adsorbed pads to plastic supports with the nitrocellulose membrane (2) (see Figure 1B) or sample pads, and we attached adsorbed pads to plastic supports with the nitrocellulose membrane (3) (see Figure 1C).

For test strips with a longer conjugate pad (10 mm) (4) (see Figure 1D), GNP–antibody conjugates (OD_520_ = 4; 1.6 mL per mm of strip width) were dried in a longer conjugate pad (10 mm) and attached with the adsorbed pads to plastic supports with the nitrocellulose membrane.

After the membranes were assembled, we used an Index Cutter-1 (A-Point Technologies, Gibbstown, NJ, USA) to cut the multi-membrane composites into 3.5 mm-wide strips. We used an FR-900 continuous band sealer (Dingli Packing Machinery, Wenzhou, China) to seal the strips hermetically into bags composed of laminated aluminum foil with silica gel as a desiccant.

### 2.14. LFIA Performance

The assay was performed at room temperature. The test strip with a conjugate pad was vertically submerged in the tested sample for 1.5 min before it was removed and placed on a horizontal surface.

For test strips with pre-mixing, we carried out two different experiments. First, the test strips were dipped into wells with a sample volume of 64 µL, and then mixed with 6 µL of GNP conjugate (OD_520_ = 4). Second, the test strips were dipped into wells containing only the sample. After a period of eight minutes, when all of the liquid was transported through capillary movement in the nitrocellulose membrane, we removed the test strips and dipped them in a solution with the GNP conjugate (70 µL, OD_520_ = 0.32).

The formations of the colored zones were visually detected 10 min after the test strips were immersed. The visual LOD of the LFIA was defined by PVY concentration when the test line appeared. In the quantitative analysis, the test strips were scanned using a Canon 9000F Mark II scanner (Canon, Tokyo, Japan), and the digital images were analyzed using a TotalLab TL120 software (Nonlinear Dynamics, Newcastle upon Tyne, UK).

### 2.15. Statistical Analysis

Each ELISA and LFIA experiments were performed at least in triplicate. The results were expressed as the mean of the data. The error bars on the calibration curves presented standard deviation.

## 3. Results and Discussion

### 3.1. Characterization of the Antibodies Using Sandwich ELISA

**Figure 2:**
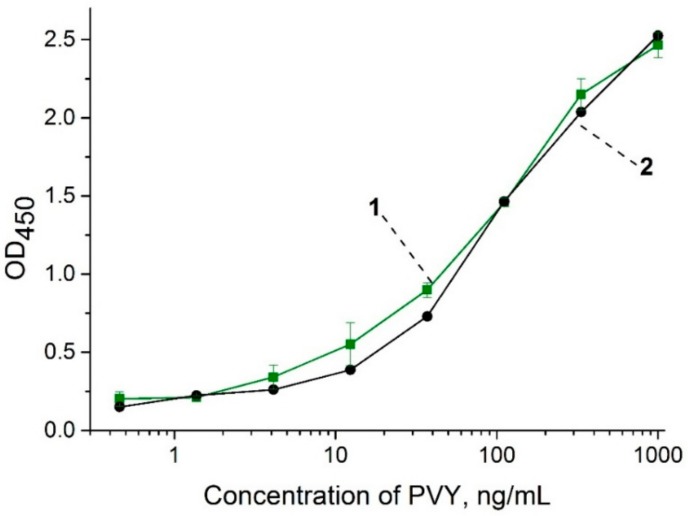
Calibration curves of ELISA for the detection of the veinal necrosis strain of PVY (PVY^N^) in extracts of healthy potato leaves (1) and buffer (2).

The obtained polyclonal antibodies specific to PVY^N^ (anti-PVY) were characterized using sandwich ELISA, which was carried out in different concentrations of the PVY^N^ in PBST and leaf extracts (Figure 2). The results showed a strong recognition of the antibodies against PVY^N^ with slight differences in PBST and leaf extracts. The LOD for PVY^N^ in both the buffer solution and the extracts was 4 ng∙mL^−1^.

There are three main PVY strains: the ordinary or common strain (PVY^O^), the C strain (PVY^C^), and the veinal necrosis strain (PVY^N^). However, genetic and serological differences divide PVY on two types of CP, namely N and O serotypes [35]. Therefore, we determined the cross-reactivity of the antibodies against PVY^O^. The cross-reactivity between PVY^N^ and PVY^O^ was determined to be 71.5% in the extracts of healthy potatoes (see the calibration curve in the Electronic Supplementary Material (ESM), Figure S1). The high cross-reactivity shown in the results is related to the polyclonal origin of antibodies, which made it possible to effectively detect both serotypes of the virus. Furthermore, the anti-PVY antibodies were tested by ELISA in reaction with other potato viruses (i.e., potato virus X, M, S, A, and potato leafroll virus) (experimental plots are shown in ESM, Figure S2). In virus concentrations up to 1 µg∙mL**^−^**^1^, the registered OD_450_ values corresponded to the background signal (cross-reactivity is less than 0.5%), which confirmed the high specificity of the anti-PVY antibodies. Thus, the obtained antibodies were shown to be an effective tool for PVY recognition of the PVY strains, because CP belongs either the N or O serotypes.

### 3.2. Constant Measurements of the Interactions between Antibody and PVY

The SPR method was used to characterize the formation of PVY^N^ complexes with anti-PVY antibodies. The kinetic and equilibrium constants of the interactions were obtained using a special sandwich scheme (covalently immobilized anti-PVY antibody–PVY–anti-PVY antibody in buffer flow), which was previously found to be successful for polyvalent viruses [32]. First, the anti-PVY antibodies were immobilized covalently with a high SPR response (about 5210 RU) (see the sensogram of immobilization in ESM, Figure S3). Second, PVY was bound by the immobilized antibodies. Third, the anti-PVY antibodies in buffer flow at different concentrations were added, and the constants in the interactions were registered (ESM, Figure S4 shows a sensogram of a typical cycle for an SPR experiment). The proposed scheme provides a more native state of PVY in its interaction with anti-PVY antibodies compared with the scheme using direct PVY immobilization.

**Figure 3:**
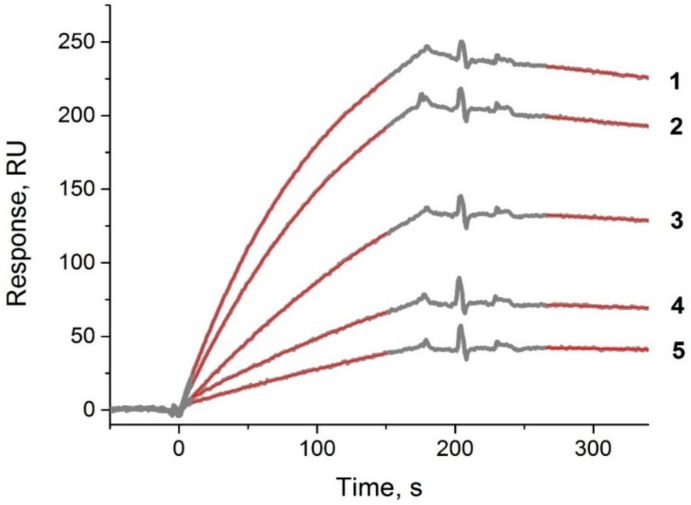
Sensograms for the binding of anti-PVY antibodies at different concentrations with PVY^N^. Experimental data are shown as thin gray lines; results of curve fittings are shown in the top layer as thick red lines. Numbers designate the concentrations of antibodies (nM): 1—330, 2—240, 3—160, 4—80, 5—50.

The obtained sensograms of the interactions between PVY^N^ and anti-PVY antibodies are shown in Figure 3. The kinetic and equilibrium dissociation constants were calculated: *k_d_* = (3.9 ± 0.4) × 10^−4^∙s^−1^, *k_a_* = (2.9 ± 0.5) × 10^4^ M^−1^∙s^−1^, *K_D_* = 1.4 × 10^−8^ M. The obtained constants characterized the interactions as not high affine. However, regarding the polyclonal antibodies specific to potato virus X with a similar *K_D_* (1.05 × 10^−8^ M), the LOD of the developed LFIA with the pre-incubation of the antibody–magnetic nanoparticle conjugate and sample for 10 min was equal to 3 ng∙mL^−1^ [22].

### 3.3. Characterization of GNPs and Its Conjugates with Antibodies

We synthesized and characterized the GNPs by TEM (data shown in ESM, Figure 5Sa,b). The GNPs had a narrow size distribution with an average diameter of 17.4 ± 1.0 nm and a form factor (i.e., the ratio of the maximum and minimum axes) of 1.1 ± 0.1. The homogeneity of the GNPs in the solution and the absence of aggregates was shown by dynamic light scattering (DLS) (data shown in ESM, Figure 5Sc), which also confirmed the dimensions of the nanoparticles (the average hydrodynamic diameter was 19.5 nm). Based on our previous experiences in the synthesis of GNP conjugates, we confirmed that spherical GNPs with diameters of 20–30 nm provided high stability to the conjugate [36,37]. Based on the characterized GNPs, we synthesized a GNP conjugate with the anti-PVY antibodies.

**Figure 4:**
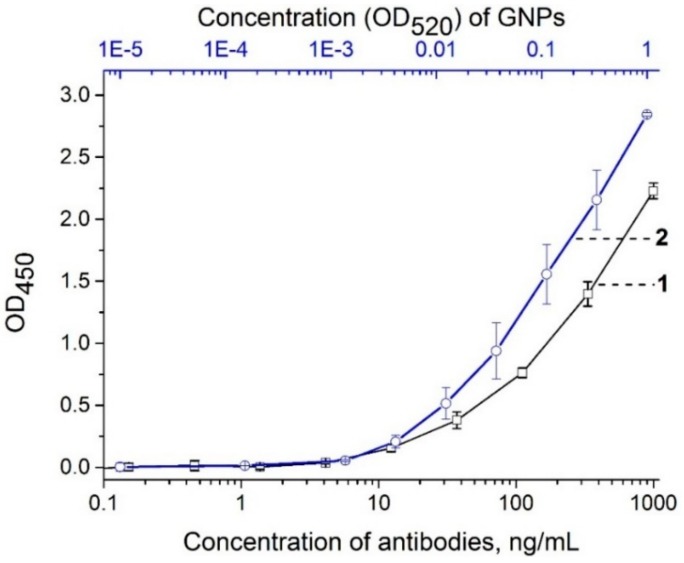
Enzyme-linked immunosorbent assay (ELISA) testing of the binding between immobilized PVY^N^ and anti-PVY antibody (curve 1), and GNP–anti-PVY antibody conjugate (curve 2).

To estimate the efficiency of antibody immobilization for the GNP conjugate, an ELISA test was performed with the formation of triple PVY^N^–GNP conjugate–anti-rabbit antibody–peroxidase conjugate complexes (see section “ELISA testing of antibody and GNP–antibody conjugates”). We compared the antigen-binding activities of the GNP–anti-PVY antibody conjugate and the unmodified anti-PVY antibodies. The results showed that the GNP–antibody conjugates with a concentration of 0.003 OD_520_ bound PVY^N^ were immobilized in the microplate wells in the same manner as the unmodified antibodies at a concentration of 12 ng∙mL^−1^ (Figure 4). Since we synthesized the conjugate using 12 μg of antibodies per 1 mL of the GNPs with an OD_520_ of 1.0, OD_520_ = 0.003 corresponds to 36 ng∙mL^−1^ of antibodies. The acceptable coincidence (36 ng∙mL^−1^ and 12 ng∙mL**^−^**^1^) of the antigen-binding activity of the conjugate and the antibody indicated that the antibodies were successfully immobilized on the GNP surface and retained their antigen-binding activity. Thus, the results showed that the GNP–antibody conjugate was appropriate for LFIA development.

### 3.4. Comparative Assessment of LFIAs at Different Schemes

**Figure 5:**
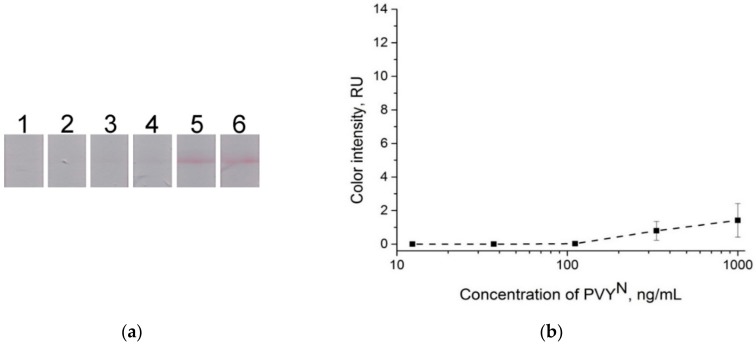
Conventional lateral flow immunoassay (LFIA). (**a**) Lateral flow strips after testing of the samples containing PVY^N^ in buffer solution, test strip 1 is negative control (0 ng**∙**mL^−1^), and test strips 1–5, PVY^N^ concentration: 12 ng**∙**mL^−1^, 37 ng**∙**mL^−1^, 111 ng**∙**mL^−1^, 333 ng**∙**mL^−1^, and 1000 ng**∙**mL^−1^, respectively. (**b**) Dependence of color intensity of the test zone on the PVY^N^ concentration.

A conventional LFIA with a conjugate pad (see Figure 1A) was first fabricated and tested for the detection of PVY^N^. Conjugate pads were attached to the test strips (as described in the Materials and Methods section) containing GNP conjugates with 4.0 OD. However, the results showed that the signals were weak, and the visual LOD was 330 ng**∙**mL^−1^ (Figure 5).

**Figure 6:**
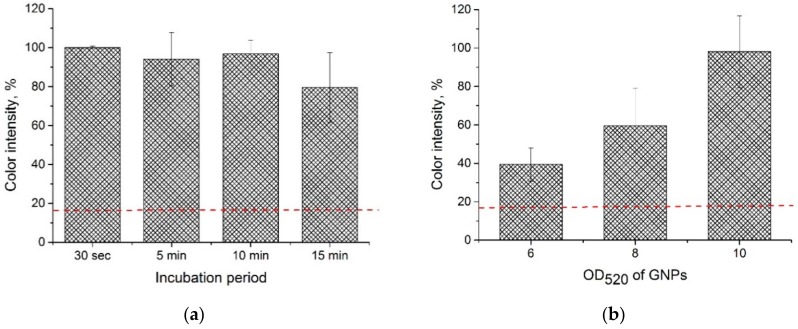
LFIA (**a**) with the addition of GNP conjugates in buffer solution and varied incubation time, (**b**) with GNP conjugates adsorbed on a conjugate pad and varied conjugate concentration. The PVY^N ^concentration in all of the test strips was 1 µg∙mL^−1^. The red dashed line corresponds to the average percentage of color intensity in test strips with a conjugate pad and OD_520_ = 4 (conventional LFIA).

Based on the results of low LOD, we had to find another way to detect PVY using LFIA. For this purpose, we used an approach in which the sample was pre-incubated with a conjugate. We conducted pre-incubation at different times. The comparison of the test strips (fabricated according to the scheme shown in Figure 1B) after pre-incubation with the test strips (fabricated according to the scheme shown in Figure 1A) without pre-incubation (Figure 6a) demonstrated an increase in the color intensity after pre-incubation. Furthermore, the results showed that even 30 s of incubation was enough to achieve the desired increase in color intensity (see Figure 6a). To shorten the duration of the analysis, we used the 30-s pre-incubation period in all of the further experiments. The results that were obtained using this approach proved to be better than the increase in the conjugate concentration in the membrane drying (conjugate pad) (Figure 6b). As shown in Figure 6b, a high signal was detected from the test strips with the attached conjugate pads on which the GNP was adsorbed and dried when we raised the OD_520_ to 10. However, at higher concentrations of the GNP conjugate (OD_520_ = 8 and 10), non-specific staining of the test zone was observed in the absence of PVY.

**Figure 7:**
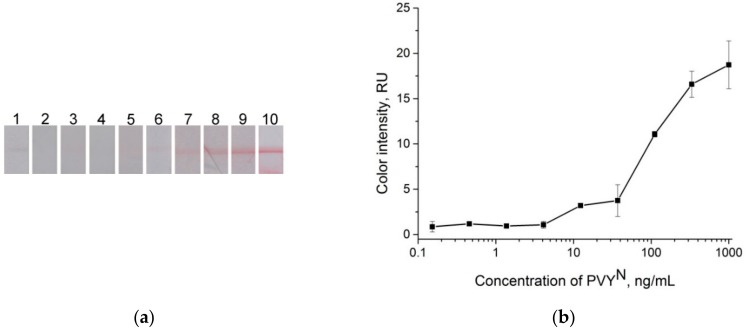
LFIA in buffer. (**a**) Lateral flow strips after testing of the samples containing PVY^N^, test strip 1 is the negative control (0 ng∙mL^−1^), and test strips 2–10, PVY^N^ concentration: 0.15 ng∙mL^−1^, 0.46 ng∙mL^−1^, 1 ng∙mL^−1^, 4 ng∙mL^−1^, 12 ng∙mL^−1^, 37 ng∙mL^−1^, 111 ng∙mL^−1^, 333 ng∙mL^−1^, and 1000 ng∙mL^−1^, respectively. (**b**) Dependence of color intensity of the test zone on the PVY^N^ concentration.

Therefore, LFIA with pre-incubation of the GNP conjugates with a sample was used to obtain the calibration curve and determine the LOD. The synthesized GNP conjugates at OD_520_ = 4 were added to the different concentrations of PVY^N^ in buffer solution without incubation. Figure 7 shows the results of the LFIA in buffer for the test strips only in the test zone. The visual LOD was 12 ng∙mL^−1^. The plotted dependence of the color intensity of the test zone on the virus concentration (Figure 7b) allowed for calculating the LOD (using the three-sigma method) as equal to 5.4 ng∙mL^−1^.

The results showed that pre-incubating the sample with the GNP conjugate decreased the LOD by 60 times compared with the conventional test strips. The obtained improvement in the LOD was higher than previously known for competitive LFIA, which showed a LOD that decreased 14 times [18,19]. This effect is significant, because it was not previously shown in the sandwich formats of LFIA. Moreover, in this study, the pre-incubation times (10 min and more) were comparable with the entire assay duration, and the benefits of LFIA as a rapid method were negated. In this study, the proposed 30-s pre-incubation period improved the results of the LFIA. Also, note that obtained result is significantly more than the basic improvement approach, namely increasing the size of nanoparticles, could provide. Thus, an increase of the GNP size, for example, from 17 nm to 33 nm, could lead to a LOD decrease up to five times, according to Safenkova et al. [38].

Subsequently, we would like to experimentally verify the main possible reasons for the improvements when using the pre-incubated conjugate.

### 3.5. Verification of the Assumptions Explaining the Improvement by Pre-Incubating the Conjugate: Negative Effect of the Sample Pad

We assumed that the improvement was associated with the insufficient interaction of the sample with the conjugate, which occurred because of the sample pad.

**Figure 8:**
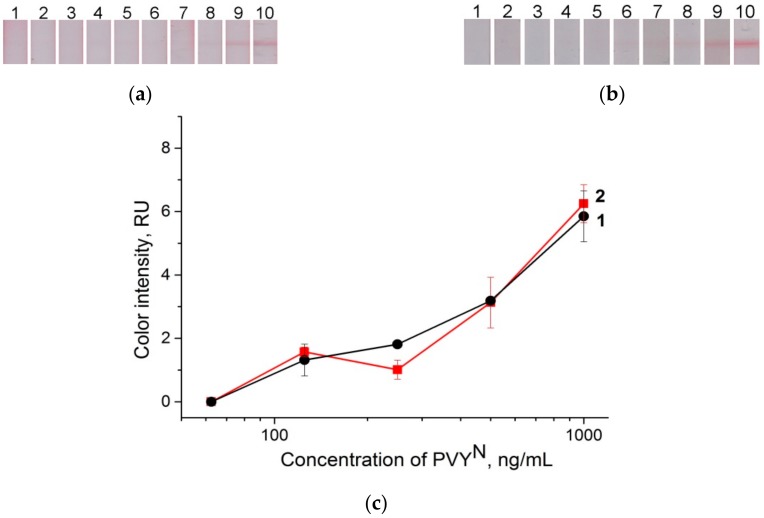
Lateral flow strips after testing of the samples containing PVY^N^ with different LFIA schemes. (**a**) Test strips with longer (1-cm) conjugate pads and without sample membrane, (**b**) LFIA with sequential addition of virus and GNP conjugate; test strip 1 is the negative control (0 ng∙mL^−1^), test strips 2–10, PVY^N^ concentration: 0.15 ng∙mL^−1^, 0.46 ng∙mL^−1^, 1 ng∙mL^−1^, 4 ng∙mL^−1^, 12 ng∙mL^−1^, 37 ng∙mL^−1^, 111 ng∙mL^−1^, 333 ng∙mL^−1^, and 1000 ng∙mL^−1^, respectively. (**c**) Dependence of color intensity of the test zone on the virus concentration, where curve 1 corresponds to the test strips in Figure 8a, and curve 2 corresponds to the test strips in Figure 8b.

To verify our assumptions, the conjugate pads with absorbed and dried GNP conjugates were enlarged to 10 mm, and then attached to the test strips without the sample membrane (see section “Preparation of lateral flow test strips” and Figure 1D). We dipped the test strips into different viral concentrations of PBST. The strips, after testing the samples containing PVY^N^, and the corresponding curve are shown in Figure 8a,c. The visual LOD of the described scheme was 125 ng**∙**mL^−1^. This result indicated that the sample membrane slightly influenced the effectiveness of the interactions between the viruses and the GNP conjugates adsorbed on the pad.

### 3.6. Verification of the Assumptions Explaining the Improvement in Pre-Incubating the Conjugate: Influence of the Sequence in the Formation of Immune Complexes 

We assumed that the formation of immune complexes would also be efficient if the complexes of the virus and the antibody adsorbed on the test zone were formed first before adding the GNP conjugate to the formation of the ternary complex (GNP conjugate–virus–antibody adsorbed on the test zone).

For verification, the test strips that were fabricated according to the scheme shown in Figure 1B were consistently dipped first into the solution with the virus, and then into the solution with the conjugate. The time intervals between the immersions were the same so that the all viral particles would bind to the antibodies in the test zone before the conjugate was added (see section “LFIA performance”). In the first scheme, the LOD was 125 ng**∙**mL^−1^ (Figure 8b,c), and the color intensity was not as high as that obtained by the LFIA method with the pre-incubation of the GNP conjugates. This result was similar to that (see Figure 5) obtained by the conventional scheme.

### 3.7. Verification of the Assumptions Explaining the Improvement in Pre-Incubating the Conjugate: Influence of the Aggregation

The results indicated that the preliminary formation of complexes of GNP conjugates with viruses promoted a better subsequent accumulation of the conjugate in the test zone in a compound of ternary complexes. A possible cause was the aggregation process (i.e., the third assumption), which is a characteristic of polyvalent interactions (the formation of branched complexes between the PVY and GNP conjugates). Previous studies showed a decreased LOD in LFIA without pre-incubation in the influenza virus (eight times) [39], potato virus X (PVX) (32 times) [40], and *Escherichia coli* O157:H7 (100 times) [41].

**Figure 9:**
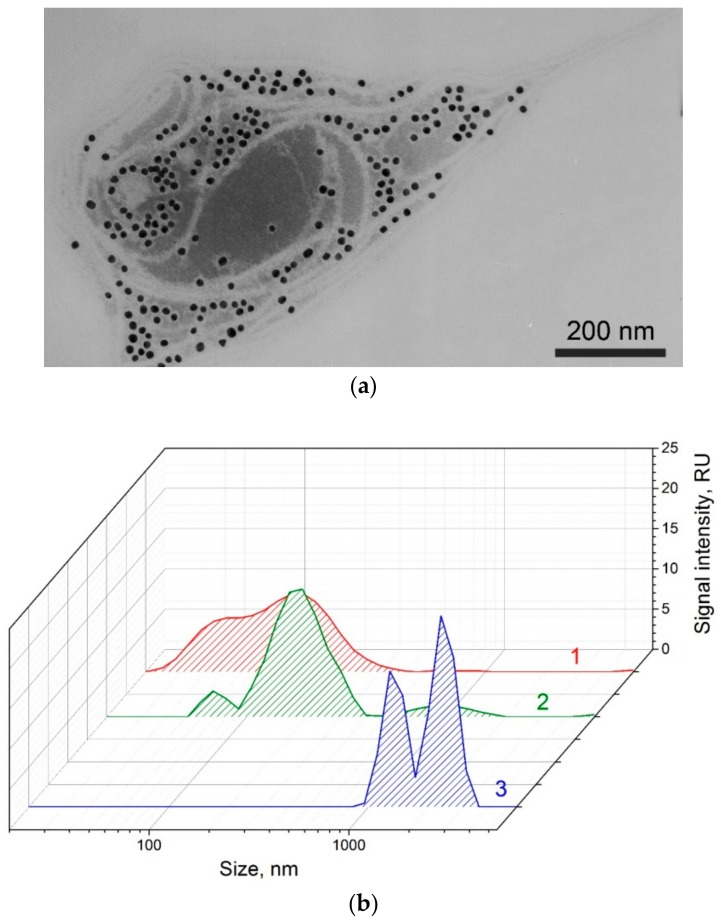
Immune complexes between PVY and GNP conjugates with antibodies. (**a**) Microphotograph by TEM. (**b**) Distribution of the hydrodynamic radii for GNP conjugates with antibodies (1), PVY (2), and PVY–conjugate complexes (3) by dynamic light scattering (DLS).

To prove the third assumption, we used TEM and DLS to study a mixture of the GNP conjugates and viruses after 30 s. Using both methods, we confirmed the presence of aggregated structures greater than 1000 nm, including a large number of GNPs (Figure 9a). The hydrodynamic radii distributions obtained by DLS showed that aggregated structures were absent in the initial preparations of the GNP conjugate (see Figure 9b, shape 1) and the virus (see Figure 9b, shape 2). However, aggregation occurred after an immunoreaction of 30-s of pre-mixing of conjugate and sample (see Figure 9b, shape 3).

Therefore, it is highly likely that the improvement caused by pre-mixing the conjugate with the sample was associated with the formation of large aggregates, which provided greater concentrations of GNPs in the test zone, greater color intensity, and hence the lower LOD of the virus.

### 3.8. Characterization of LFIA with Conjugate Pre-Mixing in Potato Leaf Extracts

**Figure 10:**
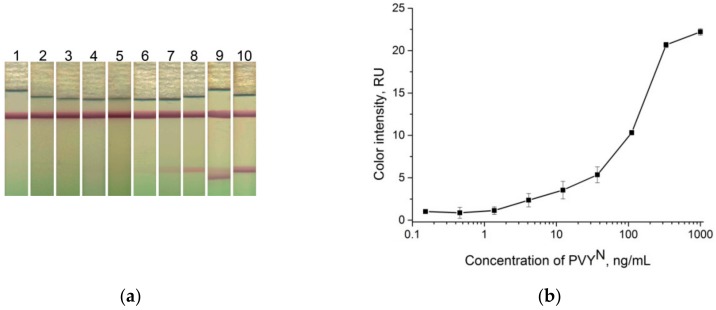
LFIA using PVY^N^–spiked plant extract, test strips without sample pad. (**a**) Test strip after analysis, test strip 1 is the negative control (0 ng∙mL^−1^), and test strips 2–10 of the PVY^N^–spiked plant extract concentration: 0.15 ng∙mL^−1^, 0.46 ng∙mL^−1^, 1 ng∙mL^−1^, 4 ng∙mL^−1^, 12 ng∙mL^−1^, 37 ng∙mL^−1^, 111 ng∙mL^−1^, 333 ng∙mL^−1^, and 1000, ng∙mL^−1^, respectively. (**b**) Dependence of color intensity of the test zone on the virus concentration.

We used the proposed LFIA with conjugate pre-mixing to test PVY^N^–spiked potato leaf extracts. Different concentrations (1000 ng**∙**mL^−1^ to 0.45 ng**∙**mL^−1^) of the PVY^N^ spiked plant extract were mixed with GNP conjugate. The test strips were dipped 30 s after mixing the reagents. The lateral flow strips after the samples containing PVY^N^ were tested, and the corresponding curves are shown in Figure 10. The LOD was 4 ng∙mL^−1^. As shown in Figure 7 and Figure 10, replacing the buffer with an extract did not worsen the PVY detection. The resulting LOD would allow for the quick and efficient diagnosis of diseases caused by PVY in non-laboratory conditions [29].

To eliminate plant extract staining on the nitrocellulose membrane, we attached a sample membrane to the test strips. The results showed less stain on the test strips compared to those without the sample pads attached (see ESM, Figure S6). The LOD was the same (4 ng**∙**mL^−1^). However, the color intensity and the contrast in the image of the potato leaf extract were higher in the test strips without sample membranes. The final test was carried out on test strips without sample membranes.

Finally, we tested infected potato leaves (four samples) using the conventional LFIA and the LFIA with pre-mixing of GNP conjugate and sample. The differences between the two approaches to applying LFIA are shown in Table 1. The LFIA based on pre-mixing the GNP conjugate and the sample better detected PVY in the samples.

### 3.9. Validation of LFIA with Pre-Mixing the Conjugate

**Figure 11:**
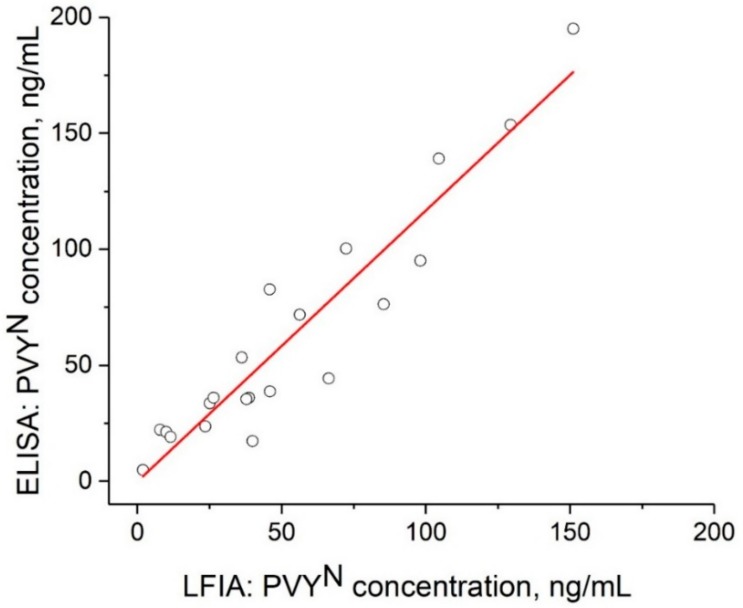
The correlation of the PVX concentration in a potato leaf extract as determined by LFIA and ELISA. The circles represent the experimental data; the solid line represents the linear approximation.

The infected potato leaf extracts were tested using the LFIA with conjugate pre-mixing and a commercial ELISA kit. In addition, 21 samples provided LFIA and ELISA data in the linear range of the calibration curves, and we calculated the PVY^N^ content of these samples. We used two linear ranges of the LFIA curve. The first is in the range from 4 ng∙mL^−1^ to 37 ng∙mL^−1^ (y = 2.1 + 0.089x, R^2^ = 0.9566); the second is in the range from 37 ng∙mL^−1^ to 330 ng∙mL^−1^ (y = 4.9 + 0.047x, R^2^ = 0.9939). The correlation between LFIA (x) and ELISA (y) was approximated with the linear function y = −0.11 + 1.17x (Figure 11). The correlation coefficient of R^2^ was 0.891, which indicated good correspondence between the data sets, and confirmed the suitability of the developed LFIA for practical use.

## 4. Conclusions

We developed the sandwich LFIA for the detection of PVY based on the pre-mixing of a GNP–antibody conjugate and the sample. We first found that pre-mixing the GNP conjugate and the sample significantly (up to 60 times) decreased the detection limit of the sandwich format of LFIA. Furthermore, we showed that the reason for the observed improvements was likely related to the formation of aggregates between the polyvalent structures (viruses and GNP–antibody conjugate) mixed in the solution. This approach could be relevant to the development of test systems for interactions with low constants (the value of *K_D_* (M) is 10^−8^), which were used in this study for PVY and antibodies specific to PVY. The test strips developed for the detection of PVY in potato leaves could be used for the non-laboratory control of diseases caused by PVY. The proposed approach is a promising solution to developing lateral flow test strips for the detection of polyvalent structures, such as viruses and bacteria.

## Figures and Tables

**Table 1:** Results of conventional LFIA and developed LFIA based on pre-mixing the GNP conjugate and the sample of potato leaf extracts. PVY^O^: ordinary or common strain of PVY.

LFIA Approach	Sample with PVY^N^	Sample with PVY^O^	Sample with PVY^N^	Sample with PVY^N^
Conventional LFIA with conjugate pad	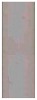	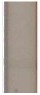	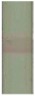	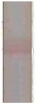
LFIA, pre-mixing GNP conjugate and sample	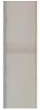	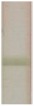	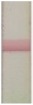	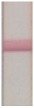

## Supplementary Materials

The following are available online at http://www.mdpi.com/1424-8220/18/11/3975/s1, Figure S1: Calibration curves of ELISA for the detection of PVY^O^, Figure S2: Calibration curves for ELISA for the detection of PVY^O^ and potato virus X, M, S, A, and potato leafroll virus, Figure S3: Sensogram of the covalent immobilization of the anti-PVY antibodies, Figure S4: Sensogram of a typical cycle for an SPR experiment on a CM5 chip, Figure S5: Transmission electron microscopy (TEM) image of GNPs and size distribution histogram. Dynamic light scattering (DLS) graph of the synthesized GNPs, Figure S6: LFIA using PVY^N^–spiked plant extract, test strips with sample pad.
