# Supplementary material for: How to Improve Sensitivity of Sandwich Lateral Flow Immunoassay for Corpuscular Antigens on the Example of Potato Virus Y?
Source: Sensors (Basel). 2018 Nov 15;18(11):3975. doi: 10.3390/s18113975 (PMC6263755; doi:10.3390/s18113975)
Supplement: Supplementary file 1 [file sensors-18-03975-s001.pdf]

Electronic Supplementary Material

How to improve sensitivity of sandwich lateral flow immunoassay for corpuscular antigens on the example of potato virus Y?

Shyatesa C. Razo[1, 2], Vasily G. Panferov[1], Irina V. Safenkova[1], Yuri A. Varitsev[3], Anatoly V. Zherdev[1], Elena N. Pakina[2], Boris B. Dzantiev[1]*

[1]A.N. Bach Institute of Biochemistry, Research Centre of Biotechnology of the Russian Academy of Sciences, Leninsky Prospect 33, 119071 Moscow, Russia

[2]Agricultural-Technological Institute, RUDN University, Miklukho-Maklaya Street 8/2, 117198 Moscow, Russia

[3]A.G. Lorch All-Russian Potato Research Institute, Kraskovo, Lorch street 23, 140051 Moscow region, Russia

*Correspondence: dzantiev@inbi.ras.ru; Tel.: +7-495-954-3142; Fax: +7-495-954-2804

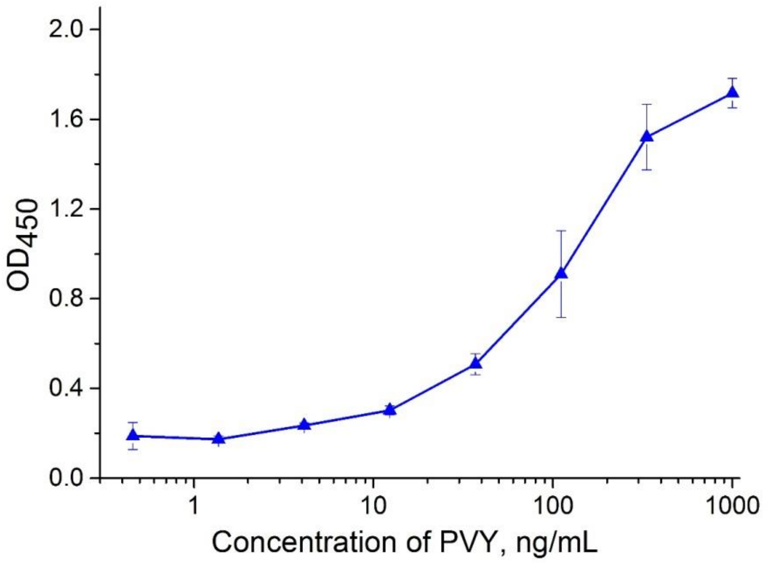

Figure S1 Calibration curve for ELISA for the detection of PVY^{O} in extracts of healthy potatoes.

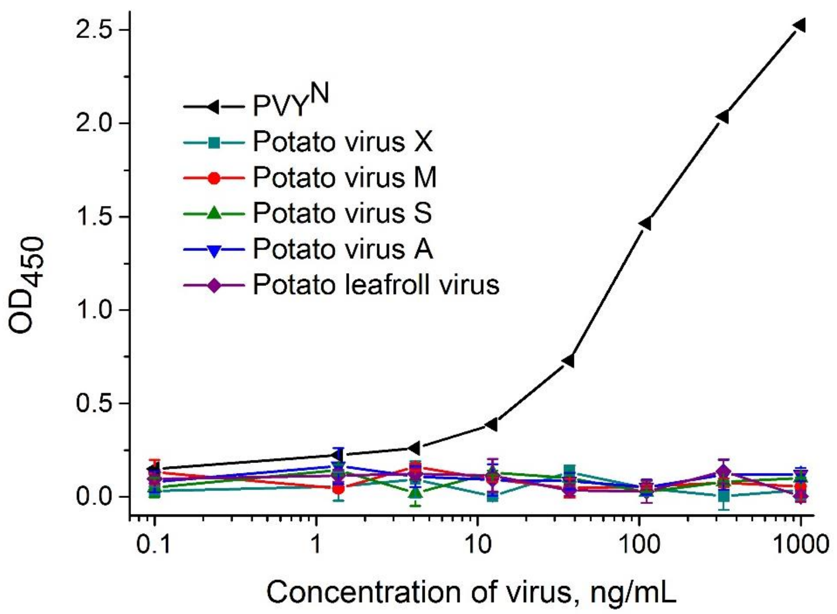

Figure S2 Calibration curve for ELISA for the detection of PVY^O and potato virus X, M, S, A, and potato leafroll virus.

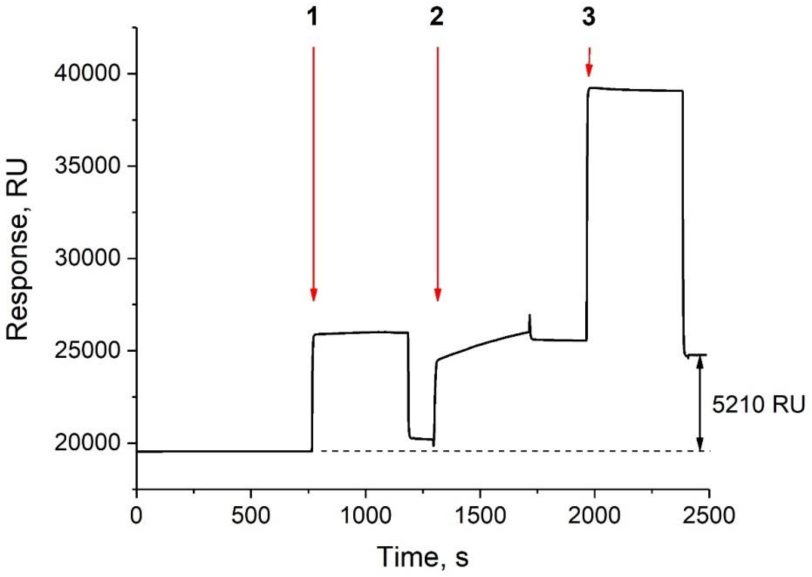

Figure S3. Sensogram of the covalent immobilization of the antibodies specific to PVY^{O} on a CM5 chip, where 1 indicates the injection of EDC and NHS, 2 indicates the injection of the antibodies, and 3 indicates the injection of ethanolamine.

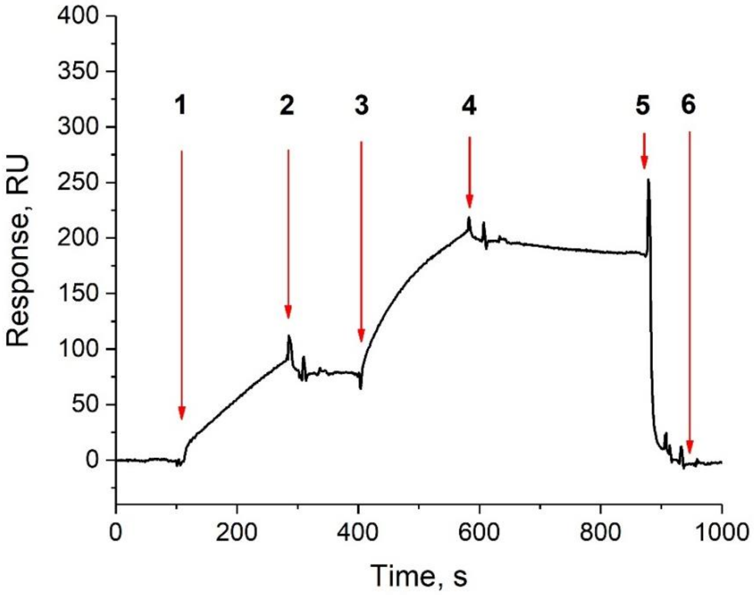

Figure S4. Sensogram of a typical cycle for an SPR experiment on a CM5 chip with covalently immobilized antibodies, where 1 indicates the injection of PVY^N at a constant concentration, 2 indicates the injection of the working buffer, 3 indicates the injection of specific (to PVY^N or PVY^O) antibodies (stage for determination of k_a), 4 indicates the injection of the working buffer (the stage of dissociation of the complex covalently immobilized antibody–PVY^N–antibody, determination of k_d), 5 indicates the injection of the buffer for destruction of the immune complex and surface regeneration up to covalently immobilized antibodies, and 6 indicates the injection of the working buffer.

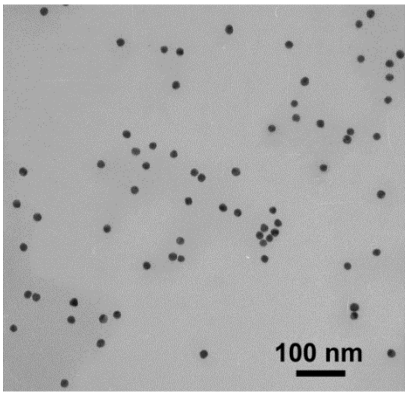

(**a**)

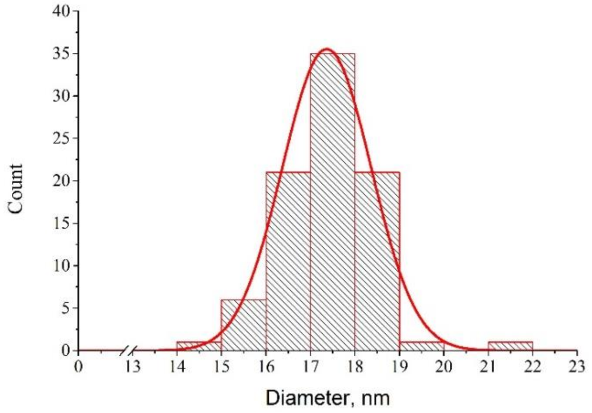

(**b**)

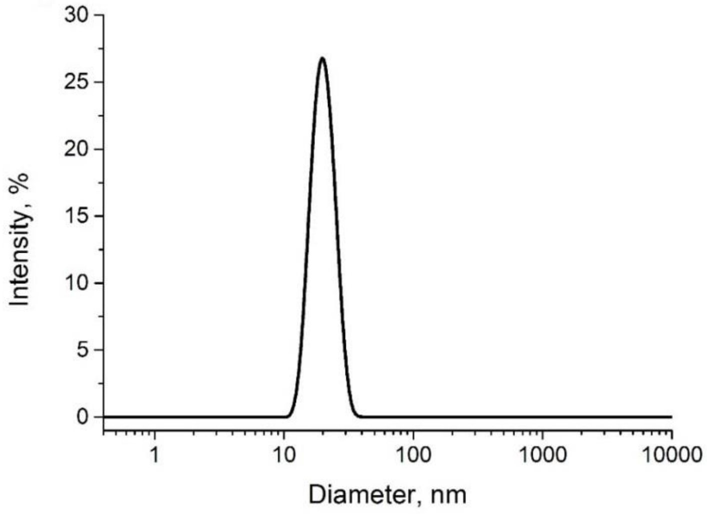

(**c**)

Figure S5. TEM image of GNPs (**a**) and size distribution histogram (**b**). DLS graph of the synthesized GNPs (**c**).

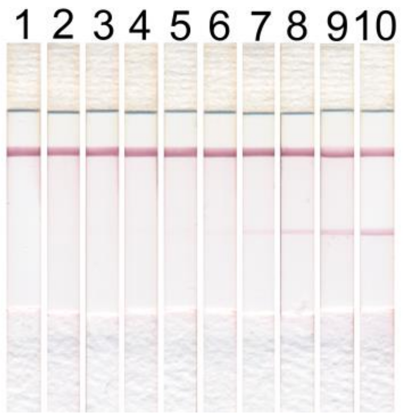

(**a**)

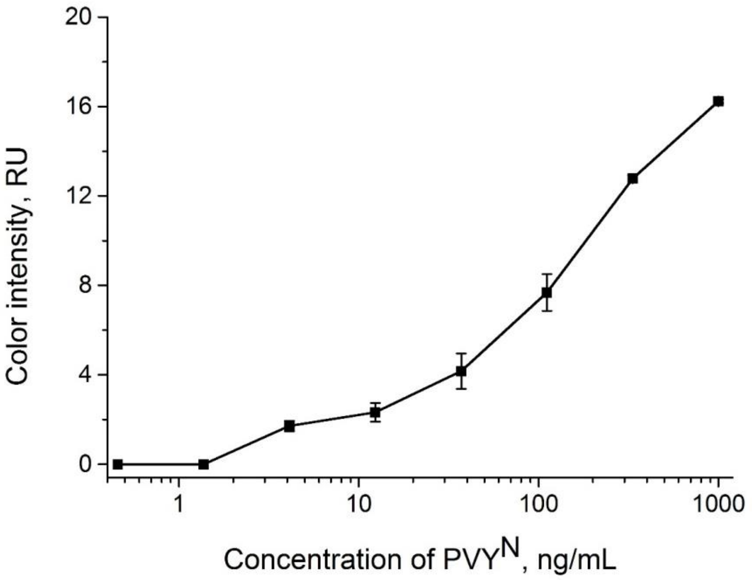

(**b**)

Figure S6. LFIA using PVY^{N}–spiked plant extract, test strips with sample pad. (**a**) Test strip after analysis, test strip 1 is the negative control (0 ng mL^{-1}) and test strips 2-10, PVY^{N}–spiked plant extract concentrations: 0.15, 0.46, 1, 4, 12, 37, 111, 333, 1000 ng mL^{-1}, respectively. (**b**) Dependence of the color intensity of the test zone on the virus concentration.
